# Listeriosis Outbreaks Associated with Soft Cheeses, United States, 1998–2014^1^

**DOI:** 10.3201/eid2406.171051

**Published:** 2018-06

**Authors:** Kelly A. Jackson, L. Hannah Gould, Jennifer C. Hunter, Zuzana Kucerova, Brendan Jackson

**Affiliations:** Centers for Disease Control and Prevention, Atlanta, Georgia, USA

**Keywords:** listeria, cheese, pasteurization, pregnancy, Hispanic, listeriosis, food safety, bacteria, United States, *Listeria monocytogenes*

## Abstract

Since 2006, the number of reported US listeriosis outbreaks associated with cheese made under unsanitary conditions has increased. Two-thirds were linked to Latin-style soft cheese, often affecting pregnant Hispanic women and their newborns. Adherence to pasteurization protocols and sanitation measures to avoid contamination after pasteurization can reduce future outbreaks.

*Listeria monocytogenes* is a widely distributed environmental bacterium that can grow at refrigeration temperatures. Infection can cause severe illness and death. Persons at higher risk for infection include older adults, persons with weakened immune systems, and pregnant women and their newborns.

Listeriosis outbreaks have been associated with refrigerated ready-to-eat foods, including hot dogs, delicatessen meats, soft cheeses, milk, and other dairy products. For soft-ripened cheeses, the risk for listeriosis per serving is estimated to be 50- to 160-fold greater for cheese made from unpasteurized milk than pasteurized milk (*1*). Pasteurization kills *L. monocytogenes*; however, milk labeled as pasteurized and dairy products made from pasteurized milk can become contaminated due to inadequate hygiene practices after pasteurization. The earliest reported listeriosis outbreak in the United States in 1985, associated with Latin-style cheese (in particular, queso fresco and cotija), resulted in 142 illnesses, 28 deaths, and 20 fetal losses (*2*). Although the cheese was labeled as made from pasteurized milk, raw milk was inadvertently introduced into the pasteurized milk.

A US retail survey of several soft cheeses (Latin-style, blue-veined, mold-ripened) from 2000–2001 detected *L. monocytogenes* in 1.3% of cheeses made from unlabeled or unpasteurized milk and 0.5% of cheeses from pasteurized milk (*3*). However, pasteurized-milk cheese is much more commonly consumed than cheese made from unpasteurized milk. In a survey of food exposures conducted in 10 US states during 2006–2007, respondents reported eating types of soft cheeses (15.3% for blue-veined cheese, 6%–11% for other soft cheeses; pasteurization status unknown) more frequently than they reported eating cheeses made from unpasteurized milk in the previous 7 days (1.6%) (*4*). We describe outbreaks linked to soft cheese (both soft-ripened and acid-coagulated–ripened cheeses), demographic characteristics of the persons affected, and possible contributing factors to help inform prevention messaging for persons at higher risk.

## The Study

Health departments in the United States electronically submit reports of foodborne disease outbreaks to the Foodborne Disease Outbreak Surveillance System (FDOSS). FDOSS captures information on etiology; implicated food; number of illnesses, hospitalizations, and deaths; and other features. We queried FDOSS for *L. monocytogenes* outbreaks (>2 cases) in the United States from 1998, when pulsed-field gel electrophoresis was first used to investigate listeriosis outbreaks, through 2014. We obtained information on fetal losses; deaths; number of cheese types; pasteurization status of milk used to make the cheese; recall issuance; and isolate subtyping from published reports (*5*–*11*), unpublished data, and food recall announcements. We considered infections in pregnant women or infants <28 days of age to be pregnancy-associated. We considered outbreaks multistate if exposure to the implicated food occurred in >1 state.

Of 58 listeriosis outbreaks reported during 1998–2014, a total of 17 (30%) were associated with soft cheese (Figure), and resulted in 180 illnesses, 14 fetal losses, and 17 deaths (Technical Appendix Table). Most patients (146, 88%) were hospitalized. Of 116 patients for whom we had information on ethnicity, 38 (33%) were Hispanic. Of 140 cases with available data, 62 (44%) were pregnancy-associated. Median outbreak size was 8 cases (range 2–34 cases). Ten outbreaks were multistate, and 16 were associated with commercial products, of which 14 involved cheeses produced in the United States. The proportion of listeriosis outbreaks linked to soft cheese made from pasteurized milk (12 outbreaks, 33%) was significantly higher during 2007–2014 than during 1998–2006 (1 outbreak, 5%; p = 0.009). Clinical isolates from soft-cheese outbreaks predominantly fell in lineage I (14 outbreaks, 82%). We found 2 sequence type (ST) and clonal complex (CC) combinations in multiple outbreaks (ST5/CC5, 5 outbreaks; ST6/CC6, 2 outbreaks), whereas other ST and CC combinations appeared in single outbreaks (e.g., ST663 or ST558).

**Figure:**
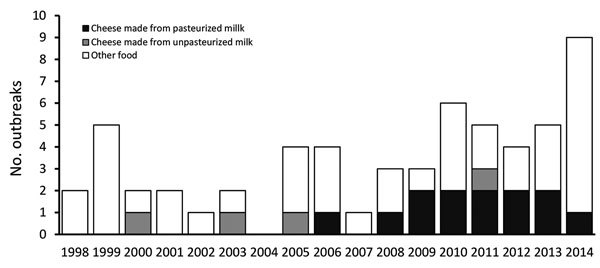
Listeriosis outbreaks associated with soft cheeses and other foods, United States, 1998–2014. The Centers for Disease Control and Prevention began pulsed-field gel eletrophoresis subtyping of clinical *Listeria monocytogenes* isolates in 1998 and launched the use of standardized interview questions in 2004; the routine use of whole-genome sequencing was introduced in 2013.

Latin-style cheeses were implicated in 11/17 (65%) outbreaks, accounting for 98 (54%) cases of listeriosis. The remaining outbreaks involved sheep’s-milk cheese, Middle Eastern– or Eastern European–style cheeses, Middle Eastern–style cheese, Italian-style cheese, blue-veined cheese, and soft-ripened cheeses (1 outbreak each). Nearly all outbreaks (13/17) resulted in recalls.

FDA inspections of cheese-making facilities associated with outbreaks found sanitation and hygiene deficiencies (e.g., roof leaks over manufacturing equipment, an open sewer vent in a manufacturing room, and food-contact aprons stored in restrooms) (*7*–*9*); pest infestations (e.g., cockroaches, flying insects) (*8*); failure to hold food at proper temperature (*8*); and presence (*8*,*11*,*12*) or persistence of *L. monocytogenes* in environmental niches of processing plants (*9*).

## Conclusions

Consumption of contaminated soft cheese made under unsanitary conditions continues to be a common cause of listeriosis outbreaks in the United States. Multiple types of soft cheeses have been implicated in outbreaks, with most outbreaks linked to Latin-style soft cheese. These outbreaks disproportionately affect Hispanic pregnant women and their neonates, a group with 24 times higher risk for listeriosis than that of the general US population (*13*). The proportion of listeriosis outbreaks caused by consumption of soft cheese made from pasteurized milk has increased in recent years. Reasons for the increase may include the growing US Hispanic population (which increased from 11% in 1998 to 17% in 2014 [*14*]); a 2.5-fold increase in per capita consumption of cheese from 1980 to 2013 (*15*); consumer demand for certain types of cheeses; and an increase in the number of small producers, some of which had sanitary deficiencies. Better outbreak detection due to improved molecular subtyping and epidemiologic methods have resulted in a greater number of solved outbreaks; however, we did not observe a similar increased proportion of outbreaks linked to other foods during the same period. This finding suggests that changes in outbreak detection are unlikely to be the only contributor.

Despite the much higher risk for listeriosis per serving of cheese made from unpasteurized rather than pasteurized milk, during the study period, only about one quarter (4/17) of all outbreaks were linked to consumption of soft cheese made from unpasteurized milk. This result may be due, in part, to public health messages advising consumers at higher risk for listeriosis not to eat these cheeses.

For instances in which information was available, we noted environmental contamination and sanitation deficiencies in all outbreaks associated with cheese made from pasteurized milk. Although some of these deficiencies were unlikely to contaminate cheese directly, they indicate a lack of attention to sanitation and hygiene. This finding highlights the importance of robust sanitation and *L. monocytogenes* monitoring programs for cheese manufacturers.

Consumers, particularly persons at high risk for listeriosis, are advised to avoid unpasteurized milk and dairy products made from unpasteurized milk. Soft cheeses made with pasteurized milk, including commercial cottage cheese, cream cheese, and processed mozzarella, are generally considered safe. However, some soft cheeses made with pasteurized milk, particularly Latin-style soft cheeses, have been produced in facilities with improper processing conditions, resulting in *L. monocytogenes* contamination. Consumers cannot evaluate the conditions under which a cheese was made on the basis of labeling or other attributes of the product. We advise persons at higher risk for listeriosis (the elderly, persons with immunocompromising conditions, and pregnant women) to carefully consider whether to consume Latin-style and other soft cheeses implicated in previous outbreaks.

Technical Appendix Additional information about listeriosis outbreaks associated with soft cheeses, United States, 1998–2014.
