# Supplementary material for: Listeriosis Outbreaks Associated with Soft Cheeses, United States, 1998–2014
Source: Emerg Infect Dis. 2018 Jun;24(6):1116–8. doi: 10.3201/eid2406.171051 (PMC6004860; doi:10.3201/eid2406.171051)
Supplement: Technical Appendix — Additional information about listeriosis outbreaks associated with soft cheeses, United States, 1998–2014. [file 17-1051-Techapp-s1.pdf]

Article DOI: https://doi.org/10.3201/eid2406.171051

Listeriosis Outbreaks Associated with Soft Cheeses, United States, 1998–2014

Technical Appendix

Technical Appendix Table. Characteristics of listeriosis outbreaks associated with soft cheese, United States, 1998–2014*

Year	State	Implicated cheese†	No. infections	No. hospitalized	No. fetal losses	No. deaths	No. (%) Hispanic	No. (%) pregnancy associated	Recall	Made from pasteurized milk	Issues or observations	Serotype /Lineage / Sequence Type (ST) / Clonal Complex (CC)‡
2000	North Carolina	Latin-style cheese	12	12	5	0	12/12 (100)	10/12 (83)	N/A§	No	Made from contaminated unpasteurized milk†	4b / I / ST558 / CC type ST558
2003	Texas	Latin-style cheese	12	12		1			N/A¶	No	Unknown	4b / I / ST1 / CC1
2005	Texas	Latin-style cheese	12	12		0			No	No	Unknown	1/2b / I / Undetermined / Undetermined
2006	Oregon	Sheep’s milk cheese	3	2	0	1	0/2 (0)	0/2 (0)	No	Yes	Unknown	4b / I / ST4 / CC4
2008	Multistate	Latin-style cheese	8	8	2	0	8/8 (100)	7/8 (88)	Yes	Yes	Inadequate separation of processing rooms (*1*)	1/2a / II / ST1216 / CC11
2009	Multistate	Latin-style cheese	18	11	0	0	10/11 (91)	9/11(82)	Yes	Yes	Unknown	4b / I / ST663 / CC type ST663
2009	Multistate	Latin-style cheese	8	3	0	0	3/3 (100)	3/4 (75)	Yes	Yes	Sanitation deficiencies<br>Failure to hold food at proper temperature<br>Failure to implement measures to exclude pests<br>Lack of appropriate training in food handling and food protection for food handlers and supervisors (*2*)#	1/2b / I / ST5 / CC5
2010	Multistate	Middle Eastern, Eastern European,	34	32	1	4	3/33 (9)	6/33 (18)	Yes	Yes	Environmental contamination with	4b / I / ST6 /CC6

Year	State	Implicated cheese†	No. infections	No. hospitalized	No. fetal losses	No. deaths	No. (%) Hispanic	No. (%) pregnancy associated	Recall	Made from pasteurized milk	Issues or observations	Serotype /Lineage / Sequence Type (ST) / Clonal Complex (CC)‡
2011	Michigan	Mediterranean, and Latin-style cheeses Middle Eastern-style cheese	2	2	1	1	0/2 (0)	1/2 (50)	Yes	Yes	*Listeria monocytogenes* (*3*) Environmental contamination with	1/2b / I / ST5 / CC5
2011	New Jersey	Latin-style cheese	2	2	0	0	1/1 (100)	2/2 (100)	Yes	Yes	*Listeria monocytogenes* Environmental contamination with	1/2b / I / ST5 / CC5
2011	Multistate	Blue-veined cheese	15	1	0	1	0/14 (0)	0/15 (0)	Yes	No	*Listeria monocytogenes* Unknown	4b / I / ST554 / CC554
2012	Multistate	Italian-style cheese (Ricotta salata)	22	21	1	4	1/14 (7)	9/22 (41)	Yes	Yes	Persistence of strains in environmental niches of processing plants in Italy (*4*)	1/2a / II / ST101 / CC101
2013	Multistate	French-style cheese (soft-ripened farmstead cheese)	6	6	1	1	1/6 (17)	1/6 (17)	Yes	Yes	Sanitation deficiencies (*5*)	4b / I / ST6 / CC6
2013	Multistate	Latin-style cheese	8	7	0	1	5/5 (100)	2/5 (40)	Yes	Yes	Sanitation deficiencies	1/2b / I / ST5 / CC5
2013	Multistate	Latin-style cheese	9	8	2	1	9/9 (100)	6/9 (67)	Yes	Yes	Environmental contamination with *Listeria monocytogenes* (*6*) Environmental contamination with *Listeria monocytogenes* (*3*)	4b / I / ST2 / CC2
2014	Washington	Latin-style cheese	3	2	0	1	3/3 (100)	1/3 (33)	Yes	Yes	Unknown	1/2a / II / Undetermined / Undetermined
Total			180	146	14	17	38/116 (33)	62/140 (44)				

*Information on fetal losses, ethnicity, and pregnancy status is not included in health department reports of foodborne disease outbreaks to the Foodborne Disease Outbreak Surveillance System (FDOSS) and therefore is missing for some outbreaks. In these instances, the denominator is the number of infections for which the characteristic was reported. CC, clonal complex; ST, sequence type.
†Latin-style cheese includes queso fresco, queso blanco, queso blando, queso cotija, queso panela, queso requesón, queso ranchero, queso Oaxaca); Middle Eastern–style cheese includes Middle Eastern string cheese, nabulsi, ackawi; Eastern European– and Mediterranean–style cheese includes feta and Bulgarian feta. A complete list of cheeses recalled in these outbreaks is available at https://www.fda.gov/Safety/Recalls/default.htm. Summaries of CDC *Listeria* outbreak investigations are available at http://www.cdc.gov/listeria/outbreaks/index.html.
‡Outbreak isolates were subject to whole genome sequencing. We sequenced Nextera XT DNA libraries on the MiSeq platform (Illumina). After quality control of raw reads, we uploaded sequences with >20x coverage in the *Listeria* whole genome multilocus sequence typing database (wgMLST) in BioNumerics 7.5, and retrieved their sequence type (ST) and lineage. We identified clonal complex (CC) using information from the MLST database of the Institut Pasteur, France; in instances where no database matches existed, we labeled outbreaks Undermined (Texas 2005, Washington 2014).
§Cheese was produced in a private home.
¶Cheese was produced at a manufacturing facility in Mexico and brought across the border for personal use.
#*L. monocytogenes* isolates from these 2 outbreaks were indistinguishable by pulsed-field gel electrophoresis. The Food and Drug Administration sought a permanent injunction against the manufacturer after the first outbreak. The owners moved the manufacturing facility to a nearby location and reopened under a new name.

References

1. Jackson KA, Biggerstaff M, Tobin-D'Angelo M, Sweat D, Klos R, Nosari J, et al. Multistate outbreak of *Listeria monocytogenes* associated with Mexican-style cheese made from pasteurized milk among pregnant, Hispanic women. J Food Prot. 2011;74:949–53. PubMed http://dx.doi.org/10.4315/0362-028X.JFP-10-536

2. US Food and Drug Administration. FDA Form 483: Quesos Mi Pueblito, LLC. 2010 Jan 20 [cited 2016 Apr 8]. http://www.fda.gov/ucm/groups/fdagov-public/@fdagov-afda-orgs/documents/document/ucm198180.pdf

3. Acciari VA, Iannetti L, Gattuso A, Sonnessa M, Scavia G, Montagna C, et al. Tracing sources of *Listeria* contamination in traditional Italian cheese associated with a US outbreak: investigations in Italy. Epidemiol Infect. 2016;144:2719–27. PubMed http://dx.doi.org/10.1017/S095026881500254X

4. US Food and Drug Administration. FDA investigation summary—multistate outbreak of *Listeria monocytogenes* linked to certain Crave Brothers Farmstead Classics Cheeses. 2014 May 16 [cited 2016 Apr 11]. http://wayback.archive-it.org/7993/20171114154927/https://www.fda.gov/Food/RecallsOutbreaksEmergencies/Outbreaks/ucm359588.htm

5. US Department of Justice. Delaware cheese company agrees to plead guilty to food adulteration charge, signs consent decree. 2016 Jan 29 [cited 2016 Apr 8]. https://www.justice.gov/opa/pr/delaware-cheese-company-agrees-plead-guilty-food-adulteration-charge-signs-consent-decree

6. Michigan Department of Agriculture & Rural Development Food and Dairy Division. Annual report fiscal year 2012 [cited 2016 Apr 8]. https://www.michigan.gov/documents/mdard/FY12_MDARD_Food_and_Dairy_Annual_report_419276_7.pdf
